# Workplace support for physicians during the COVID-19 Pandemic: Did it affect burnout?

**DOI:** 10.1186/s12913-024-11366-5

**Published:** 2024-08-03

**Authors:** Joy Melnikow, Guibo Xing, Marykate E. Miller, Sabrina Loureiro, Andrew J. Padovani, Robin Whitney, Richard L. Kravitz

**Affiliations:** 1 Department of Family and Community Medicine, Center for Healthcare Policy and Research, University of California, 4860 Y St. Suite 2300, DavisSacramento, CA 95817 USA; 2 Center for Healthcare Policy and Research, University of California, Davis, Sacramento, CA USA; 3 Center for Global Health, Weill Cornell Medicine, New York, NY USA; 4 JBS International, Inc, San Mateo, CA USA; 5 The Valley Foundation School of Nursing, San Jose State University, San Jose, CA USA; 6 Division of General Internal Medicine, University of California, Davis, Sacramento, CA USA

**Keywords:** COVID-19, Frontline physicians, Physician burnout, Workplace support, Physician wellbeing

## Abstract

**Background:**

A concern before 2020, physician burnout worsened during the COVID-19 pandemic. Little empirical data are available on pandemic workplace support interventions or their influence on burnout. We surveyed a national sample of frontline physicians on burnout and workplace support during the pandemic.

**Methods:**

We surveyed a stratified random sample of 12,833 US physicians most likely to care for adult COVID-19 patients from the comprehensive AMA Physician Professional Data ™ file. The sample included 6722 primary care physicians (3331 family physicians, 3391 internists), 880 hospitalists, 1783 critical care physicians (894 critical care physicians, 889 pulmonary intensivists), 2548 emergency medicine physicians, and 900 infectious disease physicians. The emailed survey elicited physicians’ perceptions of organizational interventions to provide workplace support and/or to address burnout. Burnout was assessed with the Professional Fulfillment Index Burnout Composite scale (PFI-BC). Proportional specialty representation and response bias were addressed by survey weighting. Logistic regression assessed the association of physician characteristics and workplace interventions with burnout.

**Results:**

After weighting, respondents were representative of the total sample. Overall physician burnout was 45.4%, significantly higher than in our previous survey. Open-ended responses mentioned that staffing shortages (physician, nursing, and other staff) combined with the increased volume, complexity, and acuity of patients during the pandemic increased job demands. The most frequent workplace support interventions were direct pandemic control measures (increased access to personal protective equipment, 70.0%); improved telehealth functionality (43.4%); and individual resiliency tools (yoga, meditation, 30.7%). Respondents placed highest priority on workplace interventions to increase financial support and increase nursing and clinician staffing. Factors significantly associated with lower odds of burnout were practicing critical care (compared with emergency medicine) OR 0.33 (95% CI 0.12 – 0.93), improved telehealth functionality OR 0.47 (95% CI 0.23 – 0.97) and being in practice for 11 years or longer OR 0.44 (95% CI 0.19–0.99).

**Conclusions:**

Burnout across frontline specialties increased during the pandemic. Physician respondents focused on inadequate staffing in the context of caring for more and sicker patients, combined with the lack of administrative efforts to mitigate problems. Burnout mitigation requires system-level interventions beyond individual-focused stress reduction programs to improve staffing, increase compensation, and build effective teams.

**Supplementary Information:**

The online version contains supplementary material available at 10.1186/s12913-024-11366-5.

## Introduction

Already a recognized concern before 2020 [1], physician burnout worsened during the COVID-19 pandemic [2–4]. Considered to result from excessive job demands combined with limited workplace resources and chronic emotional and interpersonal job stress, burnout is associated with chronic exhaustion, inefficacy, and cynicism [5].

The job demands-resources (JDR) model conceptualizes job resources as having a buffering effect on burnout resulting from excessive job demands [6, 7]. In medicine, job demands include high work pressure and emotionally demanding interactions with patients and families, both notably increased during the pandemic [4]. Job resources include physical, psychological, social or organizational aspects of the job that are functional for achieving work goals, reducing job demands, or stimulating personal growth, learning and development. Interventions that effectively address burnout may impact not only professional satisfaction and fulfillment, but also patient safety [8]. The estimated system costs of physician burnout range from $2.6 billion to $6.3 billion each year [9].

The COVID-19 pandemic markedly increased job demands on physicians, initially in a setting of scarce resources such as personal protective equipment (PPE) and COVID testing. Infected colleagues and staff were sometimes unable to quarantine due to demands increased on those still working. Simultaneously, physicians experienced increased emotional stress due to lack of effective treatments for growing numbers of gravely ill patients and personal fears of contracting COVID-19 and infecting their families, potentially resulting in severe illness and death. Once vaccinations became widely available, frontline physicians continued to care for very ill patients who had declined vaccination. These unprecedented circumstances had high potential to increase physician burnout.

Hospital and health systems made efforts to attenuate physician stress and burnout. Healthcare workplace responses during the pandemic intended to support healthcare workers included implementing policies to reduce COVID-19 transmission by increasing access to PPE and COVID-19 testing; increasing staffing and providing more flexibility of staffing assignments; expanding telehealth; and increasing communication between administrators and physicians. Other supports focused on individual healthcare workers, including opportunities for counseling and peer support, providing other wellness resources such as meditation apps and yoga classes, access to childcare, and meals.

Little empirical data are available to document these interventions or to understand their influence on burnout. To better understand the physician perspective on how workplace support was implemented during the pandemic, in June 2022 we surveyed a national sample of frontline physicians, inquiring about the types of support implemented at their workplaces during the pandemic and evaluating their burnout levels. We examined the frequency of workplace support interventions and their association with physician burnout. We compared burnout scores in the current survey to scores from frontline physician surveys conducted earlier in the pandemic (May–June 2020 and December 2020-January 2021).

## Methods

This study was considered low risk and exempt from written informed consent by the Institutional Review Board of the University of California, Davis.

### Sample

The AMA Physician Professional Data™ includes essentially all practicing physicians (MDs and DOs) in the United States; hence random samples are nationally representative [10]. We leased a random sample of frontline physician emails from the AMA Physician Professional Data including internists, family physicians, infectious disease, hospitalists, critical care, pulmonary critical care, and emergency medicine. Physicians from smaller specialties (hospitalists, critical care physicians, infectious disease physicians and emergency medicine physicians) were intentionally oversampled to improve representation in survey responses. Responses from oversampled specialties were adjusted by weighting as described below.

The 2020 sample was expanded in 2021 from 10,000 to 15,000 emails before removal of invalid emails. After removal of invalid emails over 2 rounds of surveys in 2021 and 2022, 12,833 US physicians most likely to care for adult COVID-19 patients comprised the sample generated from the comprehensive AMA Physician Professional Data ™ file. The sample included: 6722 primary care physicians (3331 family physicians and 3391 internists), 880 hospitalists, 1783 critical care physicians (894 critical care and 889 pulmonary intensivists), 2548 emergency medicine physicians, and 900 infectious disease physicians.

The data provided included information on specialty, gender, years in practice, type of practice, and practice location for each physician in the sample. This survey, our third physician survey during the pandemic that included the Professional Fulfillment Index Burnout Composite scale (PFI-BC) [11], was conducted in June 2022. Previous surveys were conducted in June-July 2020 and December 2020 – Jan 2021. The sample for these earlier surveys has been previously described [2].

### Survey

The survey was distributed in Qualtrics (Qualtrics © 2021 Provo, UT) via email three times over three weeks. To encourage response, emailed physicians were offered the opportunity to be entered in a gift card lottery. We assessed physician burnout and elicited physicians’ perceptions of organizational interventions instituted locally to provide workplace support and/or to address burnout. The survey was developed to inquire about respondents’ current work status and changes during the pandemic. It included both multiple choice questions with write-in options and narrative, open-ended questions. Burnout was assessed with the PFI-BC, a validated, open access measure of physician burnout. The PFI-BC averages the work exhaustion and interpersonal disengagement scales; respondents are asked to rate the items over the previous two weeks. Burnout was defined by a composite score of greater than 1.4 (calculated as the summed score of the 10 items comprising these two scales divided by 10) [12]. Multiple choice questions inquired about a range of options for workplace support that might have been implemented during the pandemic and which interventions at the time of the survey respondents felt should be continued or implemented. Options for interventions listed in the survey were based on prior literature [1, 13–15], focus groups with frontline physicians conducted by our team early in the pandemic, informal discussions with colleagues, and the physician authors’ personal experience. A copy of the survey is available in the online appendix (Supplement attachment 1).

### Analysis

The analysis was designed to maximize sample representativeness and reduce bias. Random sampling from a comprehensive national sampling frame ensured that survey eligibility was free of selection bias. Weighting was applied to achieve representativeness and reduce non-response bias: sampling design weights adjusted the sample to be representative of the specialties in the sampling frame, and non-response weights addressed bias due to physician response self-selection. All data processing and analyses were performed with SAS 9.4 and Stata/MP 16™. After weighting, internal medicine and family medicine physicians were combined as a primary care category, and critical care and pulmonary critical care physicians were combined as critical care for analysis.

Sampling weights, based on AMA-supplied specialty categories, were calculated as the inverse of the probability of selection for each specialty. Non-response weights were constructed using entropy balancing [16], a nonparametric generalization of the propensity score weighting approach [17] to estimate the response probability of each physician in our sample based on observed characteristics: *specialty, geography, years in practice, and type of practice.*Considering that a physician's response probability is correlated with the observable characteristics in the sampling frame data, we adjusted our sample of respondents to be representative of all sampled physicians by including the response probability in the inverse probability weighting procedure. Entropy balancing, performed with the KMATCH module for Stata, constructed unit weights calibrated to match the mean, variance, and skewness of the full sample [18].

Weighted descriptive statistics were calculated; respondent characteristics were compared to the characteristics of the full sample. Logistic regression was used to analyze association of physician characteristics and workplace support interventions with burnout, adjusting for geographic variation in the COVID-19 case count and change in COVID-19 rates in the two weeks prior to survey completion for the respondent’s county, and adjusting by the respondent’s state to account for state COVID-19 policy variation. The analysis incorporated the survey data stratification, clustering, and unequal weighting among different specialties. Spearman’s correlations were used to examine correlations between types of workplace support.

Narrative responses to questions were tabulated, reviewed, and categorized by common themes.

## Results

344 responses were received. After weighting, respondents were representative of the total sample; the standard difference in means was 0, indicating perfect balance on measured characteristics. Characteristics of respondents and non-respondents are shown in Table 1. Thirty six percent were women, 23.5% were emergency medicine physicians, 46% were primary care (internists and family physicians), 17.1% were critical care (critical care and pulmonary critical care physicians), 7.3% were hospitalists, and 6.1% were infectious disease physicians. Before weighting, a slightly higher percentage of respondents were women, practicing critical care and emergency medicine, and had been in practice for 10 years or less. Roughly 5% more respondents than non-respondents worked as hospital staff, and 6% fewer respondents were internists. Thirty one percent reported changing positions during the pandemic. Among all the respondents to the series of three surveys that included the burnout measure, 47 (6.7%) responded to all three surveys.

**Table 1:** Characteristics of survey respondents and nonrespondents before weighting and weighted Balance statistics^**a**^

**Characteristics**	**Non-respondents**	**Respondents**	**Total**		
	**N (%)**	**N (%)**	**N (%)**	**Mean** ^**b**^	**Ratio** ^**c**^
**All**	12,489 (97.3)	344 (2.7)	12,833 (100)		
**Sex**					
**Female**	4315 (34.6)	124 (36.0)	4439 (34.6)	0.000	1.003
**Male**	8174 (65.4)	220 (64.0)	8394 (65.4)		
**Physician specialty**					
**Critical Care Medicine**	862 (6.9)	32 (9.3)	894 (7.0)		
**Emergency Medicine**	2467 (19.8)	81 (23.5)	2548 (19.9)	0.000	1.003
**Family Medicine**	3242 (26.0)	89 (25.9)	3331 (26.0)	0.000	1.003
**Hospitalist**	855 (6.8)	25 (7.3)	880 (6.9)	0.000	1.003
**Infectious Disease**	879 (7.0)	21 (6.1)	900 (7.0)	0.000	1.003
**Internal Medicine**	3322 (26.0)	69 (20.1)	3391 (26.4)	0.000	1.003
**Pulmonary Critical Care Medicine**	862 (6.9)	27 (7.8)	889 (6.9)	0.000	1.003
**Type of practice**					
**Hospital staff**	2956 (23.7)	99 (28.8)	3055 (23.8)		
**Office**	9353 (74.9)	235 (68.3)	9588 (74.7)	0.000	1.003
**Teaching**	180 (1.4)	10 (2.9)	190 (1.5)	0.000	1.003
**Years in practice, 10 year increments**					
**0–10 years**	2830 (22.7)	68 (19.8)	2898		
**11–20 years**	4047 (32.4)	107 (31.1)	4154	0.000	1.003
**21–30 years**	3247 (26)	89 (25.9)	3336	0.000	1.003
**31–40 years**	1717 (13.7)	57 (16.6)	1774	0.000	1.003
**More than 40 years**	639 (5.1)	23 (6.7)	662	0.000	1.003
**Census Region**					
**Northeast**	2434 (19.5)	70 (20.3)	2504		
**Midwest**	2729 (21.9)	84 (24.4)	2813	0.000	1.003
**South**	4452 (35.6)	88 (25.6)	4540	0.000	1.003
**West**	2874 (23)	102 (29.7)	2976	0.000	1.003

^a^Weighted balance is based on diagnostic output produced by the kmatch module

^b^Mean is the standard difference in means between weighted respondents and weighted non-respondents; standard difference is 0 when perfectly balanced. Standard difference in means is rounded to 3 significant digits

^c^Ratio represents the ratio of variances of weighted non-respondents to variance of weighted respondents; ratio is 1 when perfectly balanced. Ratio of variances is rounded to 3 significant digits

Overall burnout among frontline physicians, as defined by a score > 1.4 on the PFI, decreased from 37.5% in survey 1 (June 2020) to 33.6% in survey 2 (December 2020-January 2021), and then increased to 45.4% at the 3rd survey assessing burnout (June 2022). Overall, the proportion of physicians reporting burnout in the 3rd survey was significantly higher compared with the 2nd survey (odds ratio (OR) 1.67; 95% CI 1.03–2.73). Burnout scores were similar among physicians who had changed positions (mean scale score, 1.33) and those who did not (1.31).

Open-ended responses frequently mentioned that staffing shortages (physician, nursing, and other staff) combined with the increased volume, complexity, and acuity of patients during the pandemic were sources of extremely increased job demands. Respondents also noted their perceptions of increasing polarization and anger among patients. These stressors were felt across a range of specialties and settings, from large health systems to small group or individual practices. Table 2 shows illustrative physician responses related to these themes. Several respondents also noted the need for help with childcare, which had not been supported by their workplace. A few primary care respondents noted a sense of gratification from working in effective teams.

**Table 2:** Selected open-ended responses reflecting workplace sources of physician stress and burnout during the COVID-19 pandemic

Category	Response	Specialty
Staffing	*“We are chronically understaffed. As soon as we hire a new clerical person, MA, nurse, APP, two more leave. We have many open positions with no applicants at all. Our system is trying innovative solutions–like training new MAs onsite and developing an NP Fellowship program–but it seems like a drop in the bucket.”*	Primary Care
Staffing	*“Staffing is decreased. Not because of need but because of both lack of staff and willingness of the institution to pay for it. Morale is at its lowest I have ever seen in my career. There are constant cuts in hours by administrators. This is while innumerable patients are waiting to be seen for hours. There are lack of laboratory technologists, radiology technologists, nursing, physicians. The practice of medicine has become extremely arduous and depressing. This currently is not coming from Covid. It is the mindset of administrators and institutions.”*	Primary Care
Staffing	*“Nursing attrition has devastated ICU morale”*	Critical Care
Exhaustion Lack of Support	*“Everyone manages stress differently. However, no one can witness the amount of death and tragedy that has played out in our clinics and hospitals without experiencing trauma on some level. This has and will impact everyone in the system in some substantial way, be it at work or at home. Half-assed nods and hollow campaigns to foster "wellness" and "resilience" are not going to make this right.”*	Critical Care
Exhaustion	*“As an ID physician we were putting in 14h daily 7 days a week since we are only a handful of ID physicians and the number of cases were overwhelming. It would have been nice to have the administration realize this and try to increase the number of physicians who were involved in the care of COVID patients. A lot of us were burnt out as a result of the number of hours we had to put in plus the seriousness of the illness of the patients”*	Infectious Disease
Exhaustion Lack of Support	*“We are but three pulmonary-critical care physicians working days and nights. We have no non-physician support (NPs or PAs). We put in long hours, respond in the middle of the night and go at it again the following day for one-week stretches at a time…. Surge after surge, there was no support… Hospital administration characterized our requests as "unreasonable" and they maligned our personal character and professionalism…. Leadership gives lip service to burnout, but when it comes to rubber hitting the road, they retreat. I think leadership still sees burnout as an individual's shortcoming – as if that individual has a personal character flaw that makes them not as resilient. And, it's an individual's problem to solve….”*	Critical Care
Polarization and anger	*“The politicization of getting vaccines and masks resulted in angry, threatening, and intimidating behavior in the exam room when counseling patients on health recommendations. This has never happened in 30* + *years of practice.”*	Primary Care

The frequencies of workplace support interventions reported by respondents are shown in Table 3. Direct pandemic control interventions, such as increasing access to PPE, were the most common workplace support interventions, provided by 69.8% of respondents’ workplaces. Improved telehealth functionality, the next most frequent intervention, was cited by 43.4%. Individual level resiliency tools to support physicians (for example offering peer counseling, providing apps for meditation, or yoga classes) were next most frequent (30.7%), followed by workload management (26.5%). Financial bonuses, non-financial recognition and EHR improvements were least common. We found statistically significant, but low correlations ranging from 0.12 to 0.31 between the workplace support interventions (data not shown).

**Table 3.** Weighted frequency of workplace support interventions to mitigate pandemic effects on the clinical work environment

Intervention	**Example**	**N**	**%**
**Financial bonus**	Hazard pay	48	14.2
**Non-financial recognition**	Awards, public statements by leadership	48	14.2
**Workload management**	Addition of non-physician staff	90	26.5
**Improved telehealth functionality**		146	43.4
**EHR improvements**		29	8.6
**Resiliency tools**	Yoga classes	104	30.7
**Pandemic control interventions**	Increased access to PPE	235	69.8
**Other**		21	6.3

We solicited physicians’ opinions as to which interventions ought to be initiated or continued. The most frequent responses (ranked in the top three) were financial bonuses/salary increases (47.4%), adding physician staff (34.8%), and adding nonphysician staff (33.8%). Hospitalists (64.0%) and emergency medicine physicians (57.8%) most often cited financial incentives; critical care physicians (49.1%), infectious disease physicians (47.4%) and primary care physicians (39.2%) mentioned financial incentives less frequently (chi square for distribution *p* = 0.04). Very few respondents prioritized individual level resiliency resources (3.20%) or nonfinancial recognition (3.20%).

Using multivariable logistic regression, we examined the association of burnout in this survey to specialty, years in practice, workplace support interventions, and the correspondence between what support interventions physicians said would help and what they reported that they received (Table 4). The analysis adjusted for geographic variation in COVID-19 current intensity and region. Most workplace support interventions were not associated with reduced odds of burnout. These included pandemic control interventions (which were relatively common), individual level resiliency tools (somewhat common), increased financial support, non-financial recognition, electronic health record improvements, or efforts to address workload management tools (all relatively uncommon). Factors significantly associated with lower odds of burnout were practicing critical care (compared with emergency medicine) OR 0.33 (95% CI 0.12 – 0.93), improved telehealth functionality OR 0.47 (95% CI 0.23 – 0.97) and being in practice for 11 years or longer OR 0.44 (95% CI 0.19–0.99). When a respondent’s reported workplace supports corresponded to what respondents ranked as their top 3 interventions that should be initiated or continued, burnout was lower but not statistically significant: OR 0.41 (95% CI 0.14 – 1.15).

**Table 4:** Multivariable logistic regression of examining the association of physician burnout (defined as PFI burnout score ≥ 1.4) with physician and workplace characteristics

Effect	Odds Ratio	95% Confidence Limits	
**Specialty**			
** Critical care vs Emergency medicine**	**0.329**	**0.116**	**0.933**
** Hospitalist vs Emergency medicine**	0.692	0.191	2.508
** Infectious disease vs Emergency medicine**	1.649	0.449	6.063
** Primary care vs Emergency medicine**	0.777	0.361	1.672
Region			
** Northeast vs Midwest**	0.806	0.265	2.454
** South vs Midwest**	1.433	0.579	3.547
** West vs Midwest**	1.257	0.484	3.264
**Other Factors**			
** Number of Covid cases per 100 k population**	0.999	0.995	1.003
** Improved telehealth functionality**	**0.468**	**0.227**	**0.967**
** Years of practice (> 10 years vs 0–10 years)**	**0.435**	**0.189**	**0.998**
** Workplace support interventions consistent with those prioritized by physicians ** ^a^	0.414	0.149	1.15

^a^Considered consistent if any of a respondent’s top three workplace support priorities corresponded to those reported as provided by their workplace

## Discussion

In summary, we found that burnout across frontline specialties increased between early 2020 (near the onset of the Covid-19 pandemic) and June 2022. Frontline physicians reported exhaustion (the effects of which appeared to be cumulative), demoralization resulting from inadequate staffing, and perceived inadequacy of administrative efforts to mitigate problems. Another source of professional stress was patient anger fueled by polarization. System-level workplace support interventions outside of pandemic control interventions and improved telehealth functionality were relatively infrequent. Mitigating factors associated with lower odds of burnout included working in critical care, longer experience since residency and workplace provision of improved telehealth functionality. Respondents placed highest priority on workplace interventions to increase financial support and increased nursing and clinician staffing.

Our study is limited by the low response rate and the overall cross-sectional design. The random sample from a comprehensive sampling frame with known characteristics enabled us to adjust for nonresponse bias, but concern remains that responses were not fully representative of U.S. frontline physicians caring for adults. The inverse probability weighting and propensity score-based adjustments for nonresponse that we applied are standard, widely used methods to adjust samples to be representative of the target population and reduce bias [19]. The propensity-score based methods applied in our study accounted for non-response bias correlated with observed characteristics of responders and non-responders. We cannot rule out non-response bias correlated with characteristics we could not observe, however.

We obtained our physician sample early in the pandemic, before COVID-19 was known to impact substantial numbers of children and pregnant women, consequently pediatricians and obstetricians were not included in our sample and these important frontline physician perspectives are not included. Only 6.7% of respondents to the workplace support survey responded to all three surveys; hence the data are largely cross-sectional at three time points.

The COVID-19 pandemic challenged the American healthcare delivery system and degraded the health and wellbeing of U.S. physicians. Beyond the risk of death and severe illness attendant to working in healthcare settings, particularly before vaccines were available, high pandemic-related workloads and personal stressors have taken a continued toll on the emotional and psychological wellbeing of healthcare workers. Our findings of increased burnout among US frontline physicians are consistent with other research. A national survey of physicians in the United Kingdom (UK) found that 47% experienced a decline in mental health and 34% experienced a decline in physical health since the pandemic began [20]. A similar study of family medicine physicians in the United States and Canada found high levels of depersonalization (36%) and emotional exhaustion (67%) [21]. A national survey of emergency room physicians in Canada found that while 84% reported negative emotions, such as anxiety or fear, most did not seek mental health support [22]. These findings are especially concerning in light of the already high levels of burnout and emotional exhaustion among physicians that preceded the pandemic.

In the study by Linzer et al., U.S. physicians and advanced practice clinicians experienced increasing rates of burnout throughout the pandemic. [4] Consistent with the JDR model, lack of control over work and a fast-paced, chaotic environment were associated with more burnout, whereas mitigating factors such as feeling valued and experiencing good teamwork were associated with less. These findings are consistent with comments by some primary care physicians in our survey who described the benefits of belonging to strong teams with a sense of camaraderie.

There is concern that increasing burnout will result in early retirements, further contributing to the clinician workforce shortage [23–25], though data on physician retirement rates in the US during the pandemic are lacking. Our finding that longer experience in practice was associated with lower odds of burnout is consistent with a survey of family physician educators [21]. It suggests that workplace support interventions directed to the needs of younger physicians will be important to sustain the physician workforce.

The CDC, AMA, and Joint Commission on Accreditation of Hospitals (Joint Commission) among others made recommendations on supporting healthcare workers during the pandemic [26–28]. Our study directly solicited the priorities for workplace support from frontline physicians. It is clear from our respondents that system-level interventions are needed to improve staffing, increase compensation (which may add both material and symbolic value), and build effective teams. Workplace interventions directed at individual wellness may have benefits for some individuals but are perceived by other as attempts to transfer responsibility for system-level problems to individuals. Yoga classes, meditation apps, and peer counseling, commonly offered by health systems, are not perceived as solutions to the critical system-level challenges contributing to burnout.

In terms of the JDR model [7], interventions are needed to increase resources that are functional in achieving work goals, reducing job demands, or stimulating personal growth through work. It is striking that improved telehealth functionality, a system-level intervention that fits this resource definition, was associated with lower odds of burnout. One respondent commented on the need for strategic management of increased use of telehealth: “*We need more strategic thinking about managing the shift to greater virtual care. We've adapted to telehealth and portal messaging in the near term but haven't really thought about a proactive plan to manage and promote electronic care or how to rethink provider compensation, care team productivity, or future brick-and-mortar needs.”*Interventions that provide an avenue for physicians to change burdensome aspects of the EMR or to suggest other workplace improvements have been piloted [29].

Intervention research is needed to evaluate the potential benefits of system-level changes**.** Cluster-randomized trials, regression-discontinuity designs, or step-wedge designs could evaluate the impact of interventions to build effective teams and innovative staffing models. Interventions that enable physicians to devote 20% of their medical practice to a part of their work that is especially meaningful to them have been suggested to be effective [30]. Policy changes, including nurse to patient ratios (which may impact physician workload) and work hour limits and/or workload limits for physicians also deserve further research [31, 32].

Our results represent frontline physicians’ perspectives on what workplace support interventions were implemented and what is needed going forward. While health system administrators might tell a different story, the perspectives of the potential recipients of workplace support interventions are key.

Although some impacts of the pandemic on health systems and physicians may be attenuating as the high rates of hospitalization and death due to COVID-19 have decreased, physician burnout and effective workplace interventions to mitigate burnout remain important areas for research. A concern before the pandemic, physician burnout will remain a challenge going forward. Future pandemics and other serious challenges to the delivery of health care are likely, and implementation of what we learn through research about workplace interventions to support clinician wellbeing will enhance the resilience of both individuals and health care systems.

### Supplementary Information

Supplementary Material 1.

## Data Availability

Data are provided within the manuscript and supplemental file.
