# Supplementary material for: Workplace support for physicians during the COVID-19 Pandemic: Did it affect burnout?
Source: BMC Health Serv Res. 2024 Aug 3;24:888. doi: 10.1186/s12913-024-11366-5 (PMC11298071; doi:10.1186/s12913-024-11366-5)
Supplement: Supplementary file 1 — Supplementary Material 1. [file 12913_2024_11366_MOESM1_ESM.pdf]

FINAL- S4 COVID-19 Physician Survey Series

Start of Block: Introduction

Q100 Thank you for agreeing to participate in our COVID-19 physicians' experiences survey. Your participation will help us to understand the profound impact the pandemic has had on many physicians. This information will be helpful in informing both health care and health policy. We will share the aggregate results of this survey with you when they are available.

End of Block: Introduction

Start of Block: Changes to Work Situation

Q200 These next few questions inquire about your current work situation.

Q201 Are you currently seeing patients in a clinical setting?

○ Yes (1)

○ No (2)

Q202 Have you changed positions during the pandemic?

- ○ No, I have not changed positions (1)
- ○ Yes, I have changed positions, but remain in clinical practice (2)
- ○ Yes, I have left clinical practice, but continued working (3)
- ○ Yes, I have retired, but continued some clinical practice (4)
- ○ Yes, I have retired and left clinical practice (5)
- ○ Other, please describe: (6) ____

Display This Question:

If Have you changed positions during the pandemic? = No, I have not changed positions

Or Have you changed positions during the pandemic? = Yes, I have changed positions, but remain in clinical practice

Or Have you changed positions during the pandemic? = Yes, I have retired, but continued some clinical practice

Q203 Since the pandemic began, have you increased or decreased the amount of time you devote to clinical practice?

- ○ I have increased the amount of time I devote to clinical practice by at least 50% (1)
- ○ I have increased the amount of time I devote to clinical practice by 10-49% (2)
- ○ I have not had a big change in the amount of time I devote to clinical practice (less than 10% increase or decrease) (3)
- ○ I have decreased the amount of time I devote to clinical practice by 10-49% (4)
- ○ I have decreased the amount of time I devote to clinical practice by at least 50% (5)

Display This Question:

If Have you changed positions during the pandemic? = Yes, I have left clinical practice, but continued working

Or Have you changed positions during the pandemic? = Yes, I have retired and left clinical practice

Or Since the pandemic began, have you increased or decreased the amount of time you devote to clinic... = I have decreased the amount of time I devote to clinical practice by 10-49%

Or Since the pandemic began, have you increased or decreased the amount of time you devote to clinic... = I have decreased the amount of time I devote to clinical practice by at least 50%

Q204 Why have you reduced or left clinical practice?

Please select the most important reason.

- ◯ No longer fulfilling (1)
- ◯ Did not feel supported by my health care organization (2)
- ◯ I was laid off (3)
- ◯ Exhausted by the pandemic (4)
- ◯ Wanted a different type of work (5)
- ◯ Retired at the end of my career (6)
- ◯ Wanted more time for non-work activities (7)
- ◯ Other, please describe: (8) ____

End of Block: Changes to Work Situation

Start of Block: Workplace Support

Q300 These next questions address any changes made at your workplace to support you during the pandemic and which have been most helpful.

Q301
What has your workplace done to support you during the pandemic?

Please select all that apply.

- ▢ Financial bonuses or salary increase (1)
- ▢ Adding physician staff (2)
- ▢ Adding non-physician staff (3)
- ▢ Reassigning personnel to fill immediate needs (4)
- ▢ Reducing size of patient panels (5)
- ▢ Hiring additional personnel to handle patient e-portal messaging and calls (6)
- ▢ Other support services (e.g., childcare, laundry, grocery shopping) (7)
- ▢ Expanded mental health support (8)
- ▢ Adequate availability of PPE (9)
- ▢ Accessible and rapid COVID-19 testing (99)
- ▢ Structured peer support opportunities (10)
- ▢ Wellness resources (e.g., yoga, guided meditation, gym classes, wellness apps) (11)
- ▢ Non-financial recognition (e.g., certificates of recognition, small gifts, other) (12)
- ▢ Improving telehealth functionality (13)
- ▢ Strict limits on out-of-hours work (14)
- ▢ EHR improvements (15)

- [ ] Other, please describe: (16) ____

- [ ] ⊗None (98)

Page Break

Q302
As the pandemic goes into a third year, please tell us which interventions your **workplace could continue or implement (in the future)** that would be most helpful to you.

Please click and move your ***3 top choices*** *into the box on the right, in order of most helpful to less helpful.*

Helpful Workplace Interventions

______ Financial bonuses or salary increase (1)

______ Adding physician staff (2)

______ Adding non-physician staff (3)

______ Reassigning personnel to fill immediate needs (4)

______ Reducing size of patient panels (5)

______ Hiring additional personnel to handle patient e-portal messaging and calls (6)

______ Other support services (e.g., childcare, laundry, grocery shopping) (7)

______ Expanded mental health support (8)

______ Adequate availability of PPE (9)

______ Accessible and rapid COVID-19 testing (18)

______ Structured peer support opportunities (10)

______ Wellness resources (e.g., yoga, guided meditation, gym classes, wellness apps) (11)

______ Non-financial recognition (e.g., certificates of recognition, small gifts, other) (12)

______ Improving telehealth functionality (13)

______ Strict limits on out-of-hours work (14)

______ EHR improvements (15)

______ Other, please describe: (16)

⊗______ None (17)

Q303 Is there anything else you would like to tell us about workplace changes during the pandemic, or what health systems could do to improve the workplace?

____

____

____

____

____

End of Block: Workplace Support

Start of Block: PFI

Q400 The next questions ask about your feelings towards work and burnout. To what degree have you experienced the following?

Q401 During the **past two weeks**, I have felt...

	Not at all (0)	Very little (1)	Moderately (2)	A lot (3)	Extremely (4)
A sense of dread when I think about work I have to do (PFI_1)	○	○	○	○	○
Physically exhausted at work (PFI_2)	○	○	○	○	○
Lacking in enthusiasm at work (PFI_3)	○	○	○	○	○
Emotionally exhausted at work (PFI_4)	○	○	○	○	○

Q402 During the **past two weeks**, my job has contributed to me feeling...

	Not at all (0)	Very little (1)	Moderately (2)	A lot (3)	Extremely (4)
Less empathetic with my patients (PFI_5)	○	○	○	○	○
Less empathetic with my colleagues (PFI_6)	○	○	○	○	○
Less sensitive to others' feelings/emotions (PFI_7)	○	○	○	○	○
Less interested in talking with my patients (PFI_8)	○	○	○	○	○
Less connected with my patients (PFI_9)	○	○	○	○	○
Less connected with my colleagues (PFI_10)	○	○	○	○	○

End of Block: PFI

Start of Block: END

Q501 Is there anything else you would like to tell us about how the pandemic has impacted your work or your life?

____

____

____

____

____

Q502 Would you be interested in participating in a one hour online focus group or interview to discuss your experience practicing medicine during the pandemic?

Focus group participants will be eligible for an additional gift card as a token of thanks for their time.

- Yes (1)
- No (2)

Display This Question:

If Would you be interested in participating in a one hour online focus group or interview to discuss... = Yes

Q503 Thank you for your interest! Please confirm your email below:

____

End of Block: END
